# Preparation and Performance Evaluation of Duotone 3D-Printed Polyetheretherketone as Oral Prosthetic Materials: A Proof-of-Concept Study

**DOI:** 10.3390/polym13121949

**Published:** 2021-06-11

**Authors:** Ling Ding, Wei Lu, Jiaqi Zhang, Chuncheng Yang, Guofeng Wu

**Affiliations:** 1 Department of Prosthodontics, Nanjing Stomatological Hospital, Medical School of Nanjing University, Nanjing 210000, China; 141232004@smail.nju.edu.cn (L.D.); jijun@nju.edu.cn (W.L.); mf1935157@smail.nju.edu.cn (J.Z.); 2 State Key Laboratory for Manufacturing Systems Engineering, Xi’an Jiaotong University, Xi’an 710000, China; yang.chun.cheng@stu.xjtu.edu.cn; 3 Digital Engineering Center of Stomatology and Department of Prosthodontics, Nanjing Stomatological Hospital, Medical School of Nanjing University, Nanjing 210000, China

**Keywords:** polyetheretherketone (PEEK), mechanical performance, color measurement, dual-color 3D printing, prosthodontics

## Abstract

Literature has reported the successful use of 3D printed polyetheretherketone (PEEK) to fabricate human body implants and oral prostheses. However, the current 3D printed PEEK (brown color) cannot mimic the vivid color of oral tissues and thus cannot meet the esthetical need for dental application. Therefore, titanium dioxide (TiO_2_) and ferric oxide (Fe_2_O_3_) were incorporated into PEEK to prepare a series of tooth-color and gingival-color PEEK composites in this study. Through color measurements and mechanical tests, the color value and mechanical performance of the 3D printed PEEK composites were evaluated. In addition, duotone PEEK specimens were printed by a double nozzle with an interface between tooth-color and gingival-color parts. The mechanical performance of duotone PEEK with two different interfaces (horizontal and vertical) was investigated. With the addition of TiO_2_ and Fe_2_O_3_, the colors of 3D printed PEEK composites become closer to that of dental shade guides. 3D printed PEEK composites generally demonstrated superior tensile and flexural properties and hence have great potential in the dental application. In addition, duotone 3D printed PEEK with a horizontal interfacial orientation presented better mechanical performance than that with a vertical one.

## 1. Introduction

Polyetheretherketone (PEEK) is a high-performance semi-crystalline polymer with excellent biocompatibility and great processability [1]. PEEK possesses great potential as oral prosthetic materials given its lightweight and lower modulus (3–4 GPa), which makes it a suitable alternative for conventional Co-Cr alloy (230 GPa) and Ti (104 GPa) [2]. In recent decades, PEEK has been widely used to fabricate crowns and frameworks for fixed and removable prostheses by using injection molding, milling, and 3D printing [3,4]. Despite the disadvantage of comprised esthetics, PEEK has great potential for further modification of various properties [5,6,7,8]. Much research has been performed to investigate the modified PEEK materials for enhanced performance [9]. By using compounding and injection molding [10], Ma et al. reported preparation of HA/PEEK composites and the enhanced osteogenesis acquired. Han et al. investigated the carbon fiber reinforced PEEK (CFR-PEEK) composite fabricated by fused deposition modeling (FDM), and CFR-PEEK revealed better mechanical strengths than the printed pure PEEK [8]. Another research introduced electrostatically bonded PEEK composites with increased mechanical properties and osseointegration reported [11].

3D printing has a great ability to process thermoplastics such as PEEK, with high production efficiency and low material waste compared to the traditional and subtractive techniques [12]. Recent studies also proposed the dual-nozzle printing technology for 3D printing of different materials [13], which could provide technical support for dual-color 3D printing of oral prosthetic materials with tooth-like and gingiva-like colors. Currently, the application of 3D printed PEEK is relatively few, and dual-color printing of PEEK has not been reported in dental applications. Several cases have reported the successful use of 3D printed PEEK to fabricate implants, artificial ribs, and removable prosthesis frameworks [12,14,15,16]. The limitation is that the 3D printed PEEK has a brown color, which resulted in comprised esthetics and hence affects its wide application [17]. Chen et al. reported the fabrication of a speech aid prosthesis using titanium dioxide (TiO2)/PEEK framework with enhanced mechanical strength and improved esthetics. Removable dental prostheses consist of tooth-color and gingival-color parts to mimic both hard and soft oral tissues [18]. The single-color PEEK material, however, cannot imitate the tooth and gingival colors at the same time [14]. Apart from compromised esthetics, the PEEK framework requires subsequent laboratory procedures like casting and molding to make the definitive prosthesis. Conventional fabrication procedures can be time-consuming and technology demanding. Moreover, the binding interface between different parts cannot be eliminated compared to that of one-piece printing by a double nozzle.

Therefore, this study serves as a proof-of-concept and presents the preparation, dual-nozzle printing, and performance evaluation of the dual-color PEEK composites. A series of dual-color PEEK filaments were developed by incorporating various content of titanium dioxide (TiO_2_) and ferric oxide (Fe_2_O_3_) into PEEK to alter the brown color to white and pink [14]. Then, dual-color PEEK specimens were printed using a custom dual-nozzle printer (Surgeon Pro; Shaanxi Jugao-AM Technology Co., Ltd., Xi’an, China) with optimized printing parameters [19,20,21]. Furthermore, duotone PEEK specimens with different interfacial orientations were printed using dual-color PEEK filaments by a double nozzle [13]. All specimens were evaluated by observation and instrumental color measurements, and the color difference was calculated according to the classical CIE76 formula [22]. In addition, mechanical performance of the standard test specimens was investigated using a mechanical testing machine (MTS). Their tensile and flexural properties were analyzed and compared with the frequently used dental polymethylmethacrylate (PMMA). In addition, duotone specimens with different interfacial orientations were compared based on their mechanical performance.

## 2. Materials and Methods

### 2.1. Materials Preparation

The PEEK powder (VICTREX, Lancashire, UK) was mixed with nano-titanium dioxide (TiO_2_) and ferric oxide (Fe_2_O_3_) (YIPIN Bio-Tech Co., Ltd., Ningbo, China) in this study to prepare a series of white and pink PEEK composites which imitate the colors of tooth and gingiva, respectively. The PEEK composites were mixed (mix proportions shown in Table 1) by a V-type mixer at 50 rpm for 2 min and dried in an oven at 120 °C for 3 h before use.

The mixtures were then processed separately by a twin-screw extruder (YTG-20, Shannxi Jugao-AM Technology Co., Ltd., Xi’an, China) to produce continuous filaments. The filaments (1.7 mm diameter) were cooled to 50 °C and rolled onto a reel throughout the extrusion process. Subsequently, all specimens were printed using the white (D1, D2, D3) and pink filaments (G1, G2, G3), and the printing parameters were optimized (Table 2). 

### 2.2. Color Evaluation

A Chroma Meter (TS7X, 3nh, Shenzhen, China) with the CIELAB color system was used to evaluate the color of the 3D-printed PEEK. The CIELAB system is three-dimensional, where a* axis is relative to the green (-) to red (+) opponent colors, b* axis represents the blue (-) to yellow (+) opponents, and L* axis measures relative white (100) to black (0) color. The disc-shaped specimens (5 per group) were printed using the prepared filaments separately with a diameter of 15 mm and a thickness of 3 mm. Pure PEEK specimens were printed using Jugao-MT45 PEEK filaments from the same company.

**Figure 1:**
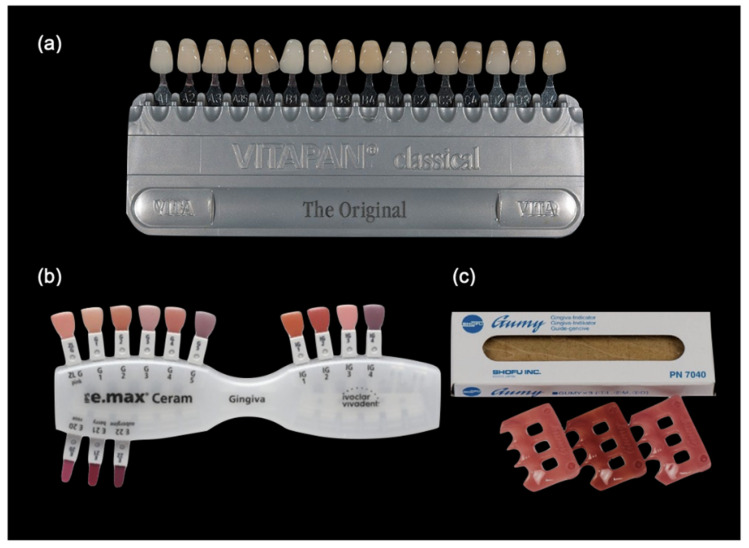
Dental shade guides. (**a**) VITA classical shade guide (Germany); (**b**) IPS e.max Ceram Gingiva Shade (Lichtenstein); (**c**) Shofu gingival shade guide (Japan).

All specimens were polished by hand with 1500, 2400, and 3000 grit sandpapers to smoothen the surface (STARCKE, Melle, Germany). The color measurements were performed with a white as well as a black background and repeated for each sample to measure the L, a*, and b* values (Table 1, Table 2). The white PEEK specimens (D1, D2, D3) were compared to the VITA Classical shade guide (Vita Zahnfabrik, Bad Säckingen, Germany) and pink specimens (G1, G2, G3) were compared to the Shofu gingiva shade guides (Shofu Dental Corp., Fukuoka, Japan) and IPS ceramic gingiva shade guide (Ivoclar Vivadent, Schaan, Liechtenstein) (Figure 1). The color difference (ΔE*) was observed and calculated according to the classical CIE76 formula [22]:

ΔE* = [(∆L*2) + (∆a*2) + (∆b*2)]½(1)

### 2.3. Mechanical Evaluation 

Mechanical properties of the 3D-printed PEEK were evaluated by tensile and flexural tests using a testing machine (EXCEED E44, MTS, Eden Prairie, MN, USA) following the manufacturer’s instruction. The specimens were designed using 3D computer-aided design (CAD) software (Dassault Systèmes SOLIDWORKS Corp., Waltham, MA, USA) according to ISO 527-2:2012 and ISO 604:2002 (Table 3) [23,24]. Standard specimens were printed using each group of the PEEK filaments separately (Group D1–D3, G1–G3).

**Figure 2:**
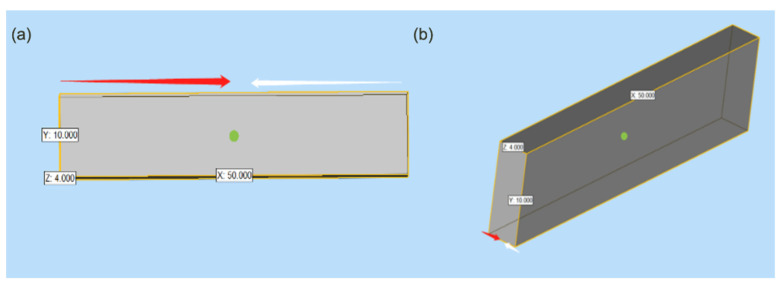
Interfacial orientations of duotone specimens. (**a**) vertical interface between white and pink parts; (**b**) horizontal interface between white and pink parts.

**Figure 3:**
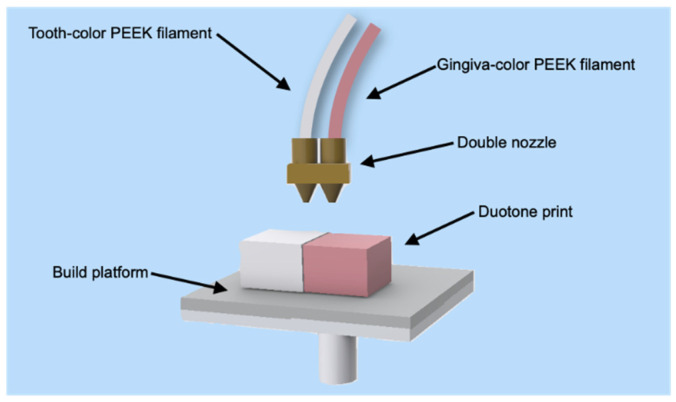
Illustration of dual-nozzle printing of the duotone specimens using white and pink polyetheretherketone (PEEK) filaments, simultaneously.

In addition, duotone specimens consisting of white and pink parts were designed with horizontal and vertical interfacial orientations (Figure 2), and these different interfacial orientations could affect the tensile and flexural properties of the 3D-printed PEEK. Thus, duotone tensile and flexural specimens (Group XY and Group Z) were printed with PEEK-D1 and PEEK-G1 filaments by double nozzle simultaneously [13], which is illustrated in Figure 3.

**Figure 4:**
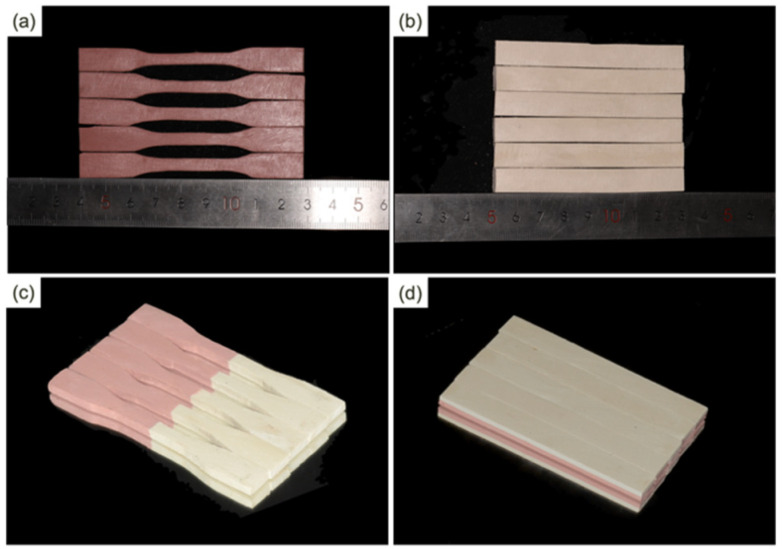
Standard test specimens. (**a**) Pink tensile specimens; (**b**) white flexural specimens; (**c**) duotone tensile specimens with vertical interfacial orientations; (**d**) duotone flexural specimens with horizontal interfacial orientations.

**Figure 5:**
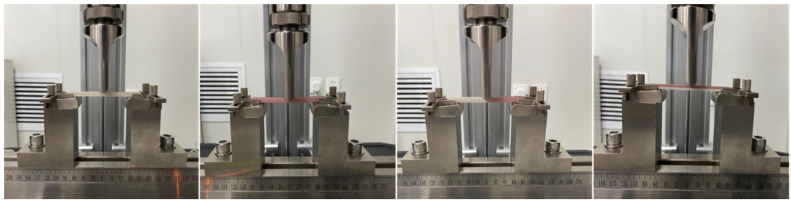
Mechanical testing using an MTS testing machine.

The specimens were polished and dried prior to testing, and 5 samples were selected for each group (Group D1–D3, G1–G3, XY, and Z). Tensile tests were performed using an MTS testing machine according to ISO 527-2:1993. Dumb-bell specimens with 90 mm test length and 4 mm thickness were tested, and the span was 60 mm. Flexural tests were performed using an MTS testing machine according to ISO 604:2002. Rectangular specimens with 80 mm test length and 4 mm thickness were tested, and the span was 69 mm. The tests were performed at 25 °C at constant speeds according to ISO standards, respectively. Figure 4 shows the tensile and flexural specimens from each category described in Table 4, including white flexural specimens, pink tensile specimens, and duotone specimens. Figure 5 shows the test equipment. The tensile and flexural properties of the specimens were obtained from the stress–strain curves and compared with that of PMMA (HUGE, Rizhao, China) by molding. Data for tensile and flexural strength and modulus are reported as the mean ± standard deviation (*n* = 5) and analyzed with one-way ANOVA for multiple comparisons using statistical software (IBM SPSS 25.0, IBM Corp, Armonk, NY, USA) (a = 0.05).

## 3. Results

### 3.1. Filament Preparation

**Figure 6:**
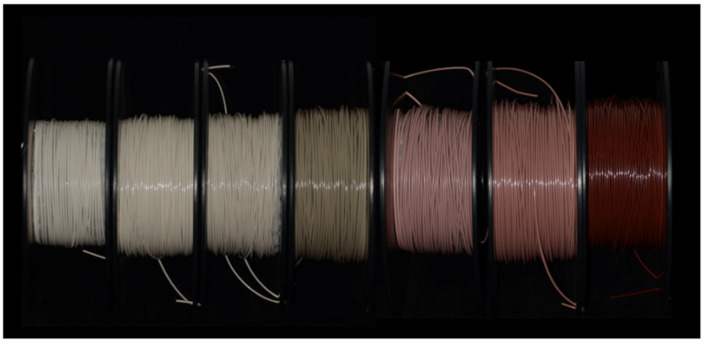
A series of dual-color PEEK filaments. From **left** to **right**: PEEK filaments with tooth-like colors (PEEK D1, D2, D3), pure PEEK filaments for contrast, and PEEK filaments with gingiva-like colors (PEEK G1, G2, G3).

Nano TiO_2_ and Fe_2_O_3_ are incorporated as functional fillers into pure PEEK by blending, and through Fused Filament Fabrication (FFF), a series of dual-color filaments with a diameter of 1.7 mm were fabricated. The filaments are divided into two categories with three tooth-like colors (PEEK D1-D3) and three gingiva-like colors (PEEK G1–G3), each as described in Table 1. Six groups of filaments were rolled onto the reels, respectively (Figure 6), and ready for use in 3D printing.

### 3.2. Color Analysis

#### 3.2.1. Results of Color Changes

**Figure 7:**
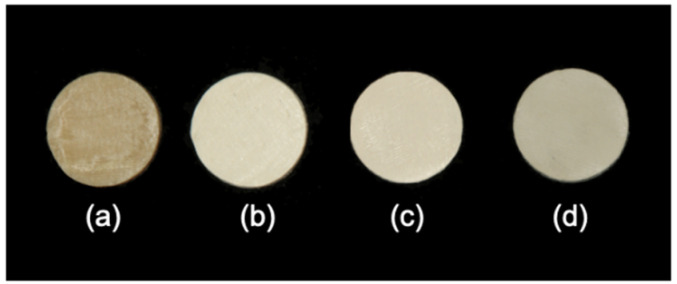
3D printed PEEK with tooth-like colors. (**a**) pure PEEK for contrast; (**b**) D1 (5% TiO_2_/PEEK); (**c**) D2 (10% TiO_2_/PEEK); (**d**) D3 (20% TiO_2_/PEEK).

**Figure 8:**
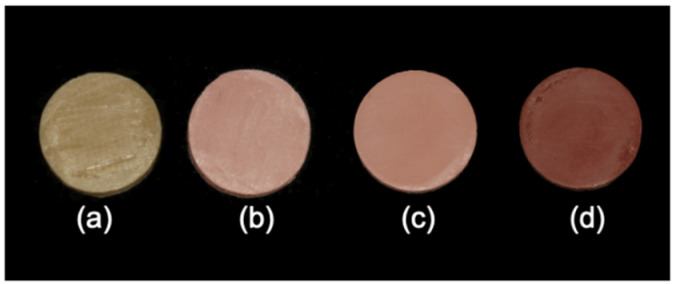
3D printed PEEK with gingiva-like colors. (**a**) pure PEEK for contrast; (**b**) G1 (20% TiO_2_/1% Fe_2_O_3_/PEEK); (**c**) G2 (10% TiO_2_/1% Fe_2_O_3_/PEEK); (**d**) G3 (1% Fe_2_O_3_/PEEK).

With addition of TiO_2_, the color of 3D printed PEEK could be altered from brown to toothlike colors (Figure 7) and becomes closer to the dental shade guide. With addition of TiO_2_ and/or Fe_2_O_3_, the color of 3D printed PEEK could be altered from brown to pink (Figure 8) and become closer to the gingiva shade guide.

#### 3.2.2. Color Coordinates and Color Differences

**Figure 9:**
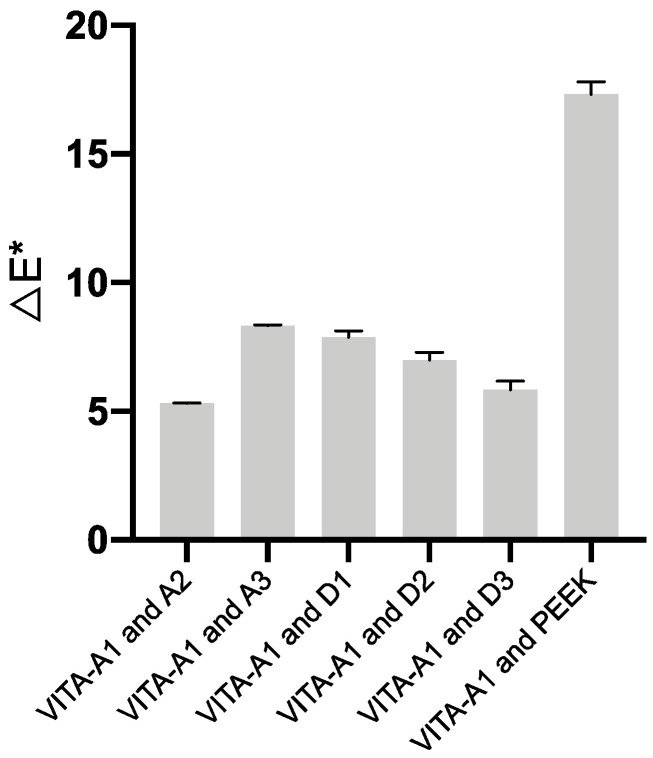
The color difference between PEEK specimens and dental shade.

**Figure 10:**
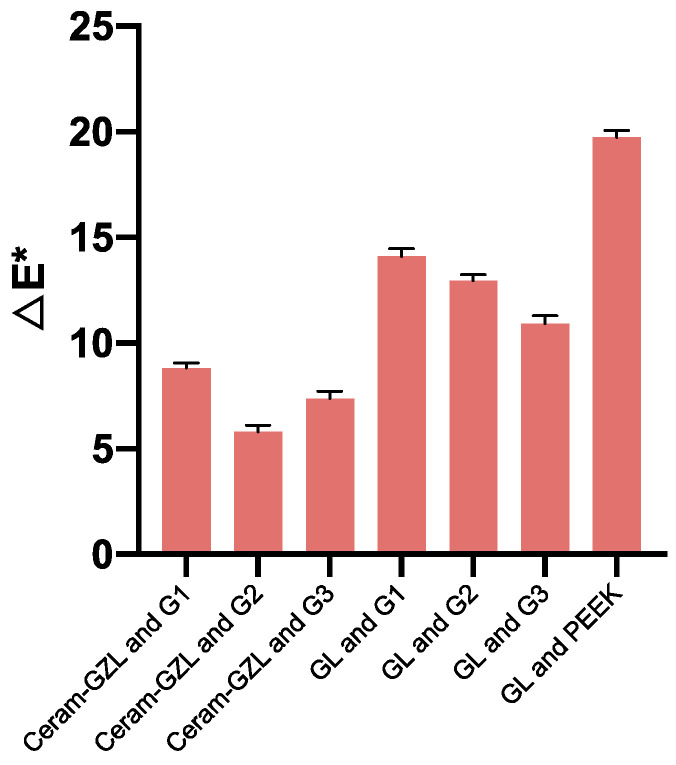
The color difference between PEEK specimens and gingiva shade.

Table 5 shows the color coordinates of white PEEK specimens (D1–D3), VITA A1-A3 shade, and pure PEEK. Table 6 shows the color coordinates of pink PEEK specimens (G1–G3), gingiva shade guides, and pure PEEK for contrast. The color difference was calculated based on the color coordinates of each category, which were consistent with visual evaluation. Figure 9 shows the results of the color difference between D1–D3 and VITA A1, and the ΔE* values varied from 5.87 (D1) to 7.92 (D3) which were smaller than the 17.36 of pure PEEK. Figure 10 shows the results of the color difference between G1–G3 and Shofu G1. The ΔE* values varied from 14.14 (G1) to 10.94 (G3) and were smaller than the 19.79 of pure PEEK. When compared to ceramic gingiva shade, G1 and G2 were close to Ceram-GZL, and G3 was close to Ceram-G4 with ΔE* values of 8.73, 5.85, and 7.73.

### 3.3. Mechanical Properties

#### 3.3.1. Tensile Performance

**Figure 11:**
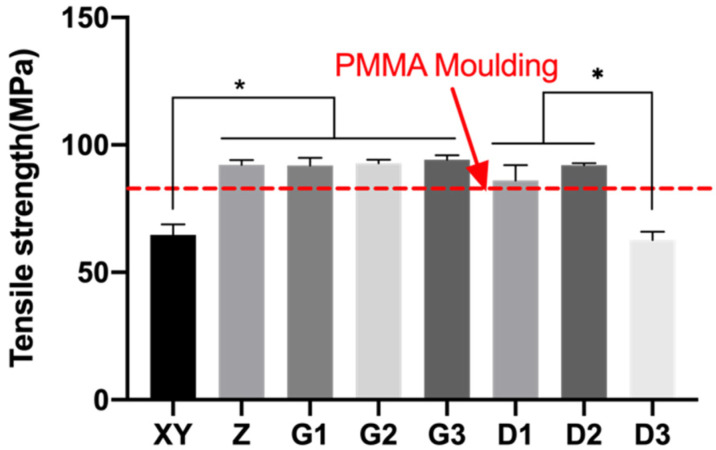
Mean and standard deviations of tensile strength of 3D-printed PEEK. * *p* < 0.05.

The mechanical properties of the 3D printed PEEK were characterized using tensile and flexural mechanical performance, which was generally better compared to PMMA. Tensile strengths of the PEEK specimens ranged between 62.74 and 94.17 MPa (Figure 11). Group D3 had the lowest strength of 62.74 MPa, while D2 had higher strength than expected. Group G1–G3 that contained 1 wt.% Fe_2_O_3_ exhibited superior tensile strength with no statistical difference observed. Group Z was not statistically different from other superior groups (D1–D2, G1–G3). Group XY had a significantly lower strength than Group Z and is likely a consequence of the vertical interface between the pink and white parts.

**Figure 12:**
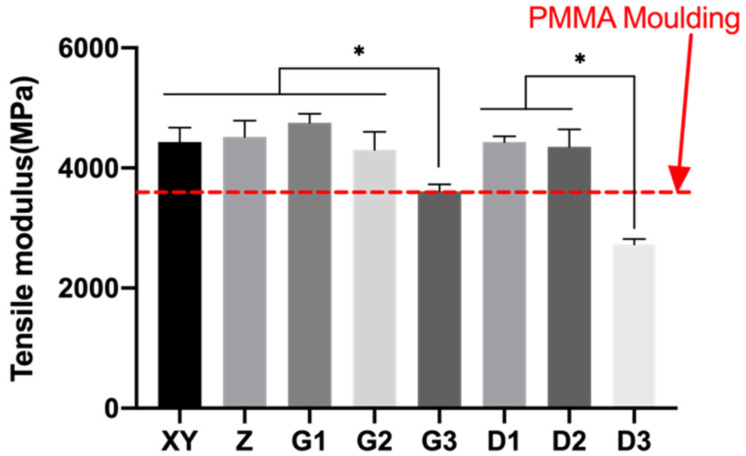
Mean and standard deviations of tensile modulus of 3D-printed PEEK. * *p* < 0.05.

Tensile moduli of the PEEK specimens ranged between 2727 and 4751 MPa (Figure 12). Group G1 had the highest tensile modulus of 4750.93 ± 153.33 MPa and pink specimens that contained more fillers exhibited higher tensile modulus. Group D3 and G3 exhibited inferior tensile modulus compared to the remaining groups and is likely due to the much lower content of fillers. The tensile modulus did not significantly differ between Group D1 (20 wt.%) and D2 (10 wt.%). Group XY was not statistically different from Group Z, although the interfacial orientations were different.

#### 3.3.2. Flexural Performance

**Figure 13:**
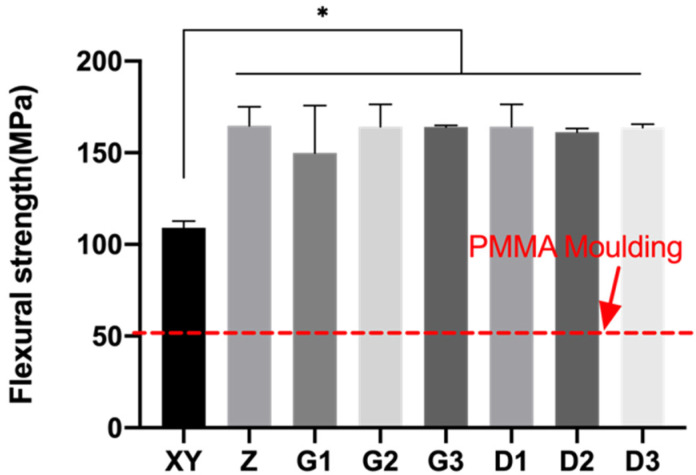
Mean and standard deviations of flexural strength of 3D-printed PEEK. * *p* < 0.05.

Flexural strengths of the PEEK specimens ranged between 109 and 164.8 MPa (Figure 13) and were significantly higher compared to that of PMMA. Group XY had the lowest flexural strength of 109.10 ± 3.61 MPa, and no statistical difference was found in the remaining groups. A possible reason could be that flexural strengths were more affected by the interfacial orientations, rather than the different content of fillers.

**Figure 14:**
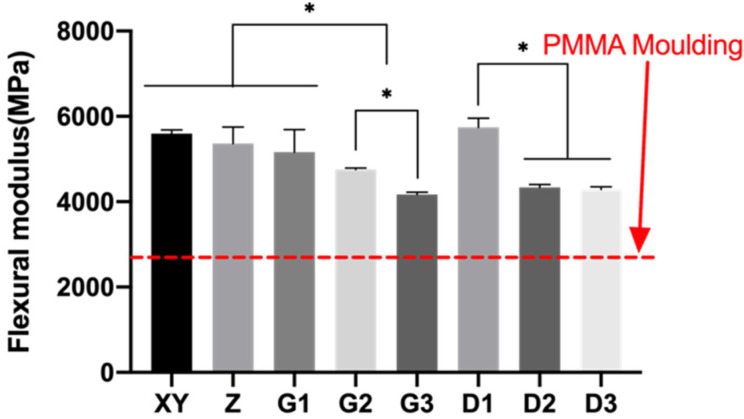
Mean and standard deviations of flexural modulus of 3D-printed PEEK. * *p* < 0.05.

The flexural moduli of the PEEK specimens ranged between 4172 and 5740 MPa, which were significantly higher compared to that of PMMA. (Figure 14). Group D1 had the highest flexural modulus of 5740.20 ± 215.93 MPa and was not statistically different from Group G1, XY, and Z, which also contained 20%wt TiO_2_. Group D2 and D3 exhibited lower flexural modulus compared to D1 possibly because of the lower content of TiO_2_, and no significant difference was observed between Group D2 (10wt.%) and D3 (5wt.%). The pink specimens that contained higher content of fillers had a higher flexural modulus (G1 > G2 > G3).

## 4. Discussion

Removable dental prostheses restore hard and soft tissues and could consist of tooth-color and gingiva-color parts to ensure both function and esthetics [2,14]. Currently, only a few commercial PEEK materials are available for molding and milling, which could not meet the need for esthetical dental restoration as well as 3D dental printing [12]. 3D printing is a kind of rapid formation (RP) technology, and it allows a customized optimization of parameters, which can be essential for the dental industries [2]. PEEK, as a thermoplastic biopolymer, possesses great thermal properties and biocompatibility, which could be suitable for 3D dental printing [12]. This study presented here is an early attempt to develop dual-color PEEK filaments for fabricating dental prostheses that consists of tooth-color and gingiva-color parts. In this preliminary study, functional fillers (nano TiO_2_ and Fe_2_O_3_) are incorporated into pure PEEK to change the brown color of 3DP PEEK to tooth-like and gingiva-like colors through Fused Filament Fabrication (FFF) [9]. Based on the filaments, duotone PEEK specimens have been successfully printed using dual-nozzle printing technology, which could provide technical support for future dual-color dental printing. One-piece fabrication can eliminate the interface between different parts and offers great efficiency and more comfort for patients compared to traditional procedures. Li et al. reported the one-piece fabrication of removable partial dentures using PEEK by milling, which showed satisfying fits [25]. This study indicates the promising application of one-piece printing using dual-color PEEK, which reduces material waste and provides improved esthetics compared to the one-piece milling. However, long-term data for the dual-color PEEK are not yet available, and continued observation is necessary to further verify the clinical outcomes. Moreover, the content of fillers and printing parameters can be flexibly adjusted and thus the properties of the printed prostheses can be tailored by further studies. The results of the color evaluation revealed that the novel PEEK composites developed in this study were closer to dental shade guides compared to pure 3DP PEEK, which provides improved esthetics for dental application. This proof of concept showed that color modification of 3DP PEEK by blending and FFF can be effective [8,9,20], although the colors obtained in this study are still limited compared to the dental shade guides. More research is required to provide more color options for 3DP PEEK with greater variety and different content of fillers and further improve the esthetics in the future.

A series of PEEK specimens with different colors have been successfully 3D printed with optimized parameters, which showed that these novel PEEK composites developed in this study are printable. Literature has reported a range of parameters for printing pure PEEK [19], and the best prints were obtained with a typical nozzle diameter of 0.4 mm and a layer thickness of 0.3 mm. Regarding the printing of other composite materials, a larger nozzle diameter (1 mm) was reported to ensure the flow rate from the nozzle [26]. Considering the values used in the literature, the parameters were selected and optimized in this study. The nozzle diameter was set at 0.4 mm to ensure the quality of prints, and the flow from the nozzle was unobstructed with the nanofillers used in this study [20,27]. The layer thickness varied from 0.1–0.2 mm to improve printing precision and reduce the void formation between layers [20]. In addition, a nozzle temperature of 420 °C, as well as a printing speed of 40 mm/s, was used considering the viscosity of the materials and the influence on the strength of prints [28]. The key parameters employed in this study can be further investigated to optimize the printing process.

Apart from the printability, it is worth noting that the PEEK composites had superior mechanical properties compared to the pure PEEK in literature, which revealed the enhancements obtained with the addition of TiO_2_ and Fe_2_O_3_ fillers in this study. Compared to rigid dental metals, the PEEK composites revealed closer tensile and flexural moduli to that of dentin, which exhibited great potential for dental application. The tensile strength reached around 90 MPa with incorporation of Fe_2_O_3_, higher than that of groups with TiO_2_ addition only. The incorporation of the fillers also increased the flexural strength, which reached above 160 MPa. The composites with higher content of fillers (5–20 wt.% TiO_2_) generally showed a higher modulus, and the highest tensile modulus of 4.75 Gpa and highest flexural modulus of 5.74 Gpa were obtained at 20 wt.%. As reported in the literature, the best mechanical performance was also reached at 20 wt.% when incorporating calcium sulfate into PEEK [29]. Other studies suggested that PEEK/hydroxyapatite composites could be enhanced at 15–30 wt.%, and moving above 20–30 wt.% could result in decreased performance and poorer prints considering the viscosity of the composites [9,30]. Therefore, the content of fillers in this study was designed to range around 5–20 wt.% under these considerations for appropriate printing process and performance. In addition, duotone specimens with a horizontal interfacial orientation generally revealed better mechanical performance compared to that with a vertical interfacial orientation. More research is required to investigate higher incorporation levels of fillers and further optimize the printing process.

## 5. Conclusions

In this preliminary study, functional fillers were incorporated into the pure PEEK to improve its esthetics for 3D dental printing. The color and mechanical performance were investigated through color evaluation and mechanical tests. The conclusions are summarized as follows:

(1) With addition of nano TiO_2_ and/or Fe_2_O_3_, white and pink PEEK filaments were developed to imitate tooth and gingiva colors. Through visual evaluation and color measurements, the color differences between the developed 3DP PEEK composites and dental shade guides were smaller compared to the pure 3DP PEEK.

(2) The tensile and flexural performance of 3DP PEEK composites was generally better than that of dental PMMA. 3DP PEEK composites had tensile and flexural moduli close to that of dentin, which exhibited great potential for dental application.

(3) Duotone PEEK specimens were printed with G1 and D1 PEEK filaments by double nozzle simultaneously. The preliminary experiments are encouraging for application in dental prostheses that consist of tooth-color and gingiva-color parts. The interfacial orientations had a significant influence on the mechanical performance of duotone prints, and duotone specimens with a horizontal interfacial orientation generally revealed better mechanical performance compared to that with a vertical interfacial orientation.

(4) 3DP PEEK composites exhibited great potential for modification and for future application in dentistry.

## Figures and Tables

**Table 1:** The mix proportions of PEEK composites.

Description	PEEK (wt.%)	TiO_2_ (wt.%)	Fe_2_O_3_ (wt.%)
PEEK-D1	80%	20%	0%
PEEK-D2	90%	10%	0%
PEEK-D3	95%	5%	0%
PEEK-G1	79%	20%	1%
PEEK-G2	89%	10%	1%
PEEK-G3	99%	0%	1%

**Table 2:** Dual-nozzle 3D printing parameters.

Description	Value
Nozzle diameter	0.4 mm
Nozzle temperature	420 °C
Nozzle pitch	87.5 mm
Bead width	0.36 mm
Layer thickness	0.1–0.2 mm
Printing speed	40 mm/s
Raster angle	±45°
Ambient temperature	RT
*z*-axis layer	0.2 mm
Infill percentage	100%

**Table 3:** Mechanical tests and standard specimens.

Tests	Size (mm)	ISO
Tensile test	90.00 × 10.00 × 4.00	ISO 527-2:2012 [23]
Flexural test	80.00 × 10.00 × 4.00	ISO 178:2019 [24]

**Table 4:** Groups in the mechanical tests and the filaments used.

Category	Groups	Filaments	Filaments
White	Group D1	PEEK-D1	/
	Group D2	PEEK-D2	/
	Group D3	PEEK-D3	/
Pink	Group G1	/	PEEK-G1
	Group G2	/	PEEK-G2
	Group G3	/	PEEK-G3
Duotone	Group XY	PEEK-D1	PEEK-G1
	Group Z	PEEK-D1	PEEK-G1

**Table 5:** Color coordinates of VITA A1–A3 and white PEEK specimens.

Groups	L*	a*	b*
VITA-A1	79.57	−1.61	13.05
VITA-A2	76.04	−0.08	16.73
VITA-A3	75.36	1.36	19.61
D1	86.34	1.28	10.14
D2	84.91	0.98	9.70
D3	83.29	1.79	10.05
PEEK	63.42	4.17	15.70

**Table 6:** Color coordinates of gingiva shade guide and pink PEEK specimens.

Groups	L*	a*	b*
Shofu-GL	52.83	13.48	1.81
Shofu-GM	50.56	13.74	3.06
Shofu-GD	44.14	12.21	3.55
Ceram-GZL	61.95	18.78	15.83
Ceram-G4	51.54	17.41	11.28
G1	65.18	16.11	8.17
G2	61.01	18.83	10.06
G3	45.26	18.70	7.73
PEEK	63.42	4.17	15.70

## Data Availability

The data presented in this study are available on request from the corresponding author.

## References

[1] Panayotov I.V., Orti V., Cuisinier F., Yachouh J. (2016). Polyetheretherketone (PEEK) for medical applications. J. Mater. Sci. Mater. Med..

[2] Qin L., Yao S., Zhao J., Zhou C., Oates T.W., Weir M.D., Wu J., Xu H.H.K. (2021). Review on Development and Dental Applications of Polyetheretherketone-Based Biomaterials and Restorations. Materials.

[3] Zoidis P., Papathanasiou I. (2016). Modified PEEK resin-bonded fixed dental prosthesis as an interim restoration after implant placement. J. Prosthet. Dent..

[4] Najeeb S., Zafar M.S., Khurshid Z., Siddiqui F. (2016). Applications of polyetheretherketone (PEEK) in oral implantology and prosthodontics. J. Prosthodont. Res..

[5] Andrikopoulou E., Zoidis P., Artopoulou I.I., Doukoudakis A. (2016). Modified PEEK Resin Bonded Fixed Dental Prosthesis for a Young Cleft Lip and Palate Patient. J. Esthet. Restor. Dent..

[6] Chen X., Ma R., Min J., Li Z., Yu P., Yu H. (2020). Effect of PEEK and PTFE coatings in fatigue performance of dental implant retaining screw joint: An in vitro study. J. Mech. Behav. Biomed. Mater..

[7] Cook S.D., Rust-Dawicki A.M. (1995). Preliminary evaluation of titanium-coated PEEK dental implants. J. Oral. Implantol..

[8] Han X., Yang D., Yang C., Spintzyk S., Scheideler L., Li P., Li D., Geis-Gerstorfer J., Rupp F. (2019). Carbon Fiber Reinforced PEEK Composites Based on 3D-Printing Technology for Orthopedic and Dental Applications. J. Clin. Med..

[9] Rodzen K., Sharma P.K., McIlhagger A., Mokhtari M., Dave F., Tormey D., Sherlock R., Meenan B.J., Boyd A. (2021). The Direct 3D Printing of Functional PEEK/Hydroxyapatite Composites via a Fused Filament Fabrication Approach. Polymers.

[10] Ma R., Fang L., Luo Z., Weng L., Song S., Zheng R., Sun H., Fu H. (2014). Mechanical performance and in vivo bioactivity of functionally graded PEEK–HA biocomposite materials. J. Sol-Gel Sci. Technol..

[11] Bastan F.E. (2020). Fabrication and characterization of an electrostatically bonded PEEK- hydroxyapatite composites for biomedical applications. J. Biomed. Mater. Res. B Appl. Biomater..

[12] Singh S., Prakash C., Ramakrishna S. (2019). 3D printing of polyether-ether-ketone for biomedical applications. Eur. Polym. J..

[13] Luo H., Tan Y., Zhang F., Zhang J., Tu Y., Cui K. (2019). Selectively Enhanced 3D Printing Process and Performance Analysis of Continuous Carbon Fiber Composite Material. Materials.

[14] Chen X., Wang F., Sun F., Zhang L., Wu G. (2020). Digital fabrication of an adult speech aid prosthesis by using a 3-dimensionally printed polyetheretherketone framework. J. Prosthet. Dent..

[15] Wang L., Liu X., Jiang T., Huang L. (2020). Three-dimensional printed polyether-ether-ketone implant for extensive chest wall reconstruction: A case report. Thorac. Cancer.

[16] Kang J., Wang L., Yang C., Wang L., Yi C., He J., Li D. (2018). Custom design and biomechanical analysis of 3D-printed PEEK rib prostheses. Biomech. Model. Mechanobiol..

[17] Zoidis P., Papathanasiou I., Polyzois G. (2016). The Use of a Modified Poly-Ether-Ether-Ketone (PEEK) as an Alternative Framework Material for Removable Dental Prostheses. A Clinical Report. J. Prosthodont..

[18] Cabello-Dominguez G., Perez-Lopez J., Veiga-Lopez B., Gonzalez D., Revilla-Leon M. (2020). Maxillary zirconia and mandibular composite resin-lithium disilicate-modified PEEK fixed implant-supported restorations for a completely edentulous patient with an atrophic maxilla and mandible: A clinical report. J. Prosthet. Dent..

[19] Deng X., Zeng Z., Peng B., Yan S., Ke W. (2018). Mechanical Properties Optimization of Poly-Ether-Ether-Ketone via Fused Deposition Modeling. Materials.

[20] Wickramasinghe S., Do T., Tran P. (2020). FDM-Based 3D Printing of Polymer and Associated Composite: A Review on Mechanical Properties, Defects and Treatments. Polymers.

[21] Li Q., Zhao W., Li Y., Yang W., Wang G. (2019). Flexural Properties and Fracture Behavior of CF/PEEK in Orthogonal Building Orientation by FDM: Microstructure and Mechanism. Polymers.

[22] Heimer S., Schmidlin P.R., Stawarczyk B. (2017). Discoloration of PMMA, composite, and PEEK. Clin. Oral. Investig..

[23] ISO 527-2:2012 Plastics—Determination of Tensile Properties—Part 2: Test Conditions for Moulding and Extrusion Plastics. https://www.iso.org/standard/56046.html.

[24] ISO 178:2019 Plastics—Determination of Flexural Properties. https://www.iso.org/standard/70513.html.

[25] Li X.X., Liu Y.S., Sun Y.C., Chen H., Ye H.Q., Zhou Y.S. (2019). Evaluation of one-piece polyetheretherketone removable partial denture fabricated by computer-aided design and computer-aided manufacturing. Beijing Da Xue Xue Bao Yi Xue Ban.

[26] Schwitalla A.D., Spintig T., Kallage I., Muller W.D. (2015). Flexural behavior of PEEK materials for dental application. Dent. Mater..

[27] Yu X., Yao S., Chen C., Wang J., Li Y., Wang Y., Khademhosseini A., Wan J., Wu Q. (2020). Preparation of Poly(ether-ether-ketone)/Nanohydroxyapatite Composites with Improved Mechanical Performance and Biointerfacial Affinity. ACS Omega.

[28] Cicala G., Latteri A., Del Curto B., Lo Russo A., Recca G., Farè S. (2017). Engineering Thermoplastics for Additive Manufacturing: A Critical Perspective with Experimental Evidence to Support Functional Applications. J. Appl. Biomater. Funct. Mater..

[29] Hughes E.A.B., Grover L.M. (2017). Characterisation of a novel poly (ether ether ketone)/calcium sulphate composite for bone augmentation. Biomater. Res..

[30] Wang L., Weng L., Song S., Sun Q. (2010). Mechanical properties and microstructure of polyetheretherketone–hydroxyapatite nanocomposite materials. Mater. Lett..

